# The Analgesic Effect on Neuropathic Pain of Retrogradely Transported *botulinum* Neurotoxin A Involves Schwann Cells and Astrocytes

**DOI:** 10.1371/journal.pone.0047977

**Published:** 2012-10-24

**Authors:** Sara Marinelli, Valentina Vacca, Ruggero Ricordy, Carolina Uggenti, Ada Maria Tata, Siro Luvisetto, Flaminia Pavone

**Affiliations:** 1 National Research Council of Italy (Cell Biology and Neurobiology Institute)/Istituto Di Ricovero e Cura a Carattere Scientifico Fondazione Santa Lucia, Rome, Italy; 2 National Research Council of Italy - Institute of Molecular Biology and Pathology, Rome, Italy; 3 Department of Biology and Biotechnologies Charles Darwin, Center of Neurobiology Research Daniel Bovet, Sapienza University, Rome, Italy; Southern Illinois University School of Medicine, United States of America

## Abstract

In recent years a growing debate is about whether *botulinum* neurotoxins are retrogradely transported from the site of injection. Immunodetection of cleaved SNAP-25 (cl-SNAP-25), the protein of the SNARE complex targeted by *botulinum* neurotoxin serotype A (BoNT/A), could represent an excellent approach to investigate the mechanism of action on the nociceptive pathways at peripheral and/or central level. After peripheral administration of BoNT/A, we analyzed the expression of cl-SNAP-25, from the hindpaw’s nerve endings to the spinal cord, together with the behavioral effects on neuropathic pain. We used the chronic constriction injury of the sciatic nerve in CD1 mice as animal model of neuropathic pain. We evaluated immunostaining of cl-SNAP-25 in the peripheral nerve endings, along the sciatic nerve, in dorsal root ganglia and in spinal dorsal horns after intraplantar injection of saline or BoNT/A, alone or colocalized with either glial fibrillar acidic protein, GFAP, or complement receptor 3/cluster of differentiation 11b, CD11b, or neuronal nuclei, NeuN, depending on the area investigated. Immunofluorescence analysis shows the presence of the cl-SNAP-25 in all tissues examined, from the peripheral endings to the spinal cord, suggesting a retrograde transport of BoNT/A. Moreover, we performed in vitro experiments to ascertain if BoNT/A was able to interact with the proliferative state of Schwann cells (SC). We found that BoNT/A modulates the proliferation of SC and inhibits the acetylcholine release from SC, evidencing a new biological effect of the toxin and further supporting the retrograde transport of the toxin along the nerve and its ability to influence regenerative processes. The present results strongly sustain a combinatorial action at peripheral and central neural levels and encourage the use of BoNT/A for the pathological pain conditions difficult to treat in clinical practice and dramatically impairing patients’ quality of life.

## Introduction

*Botulinum* neurotoxin type A (BoNT/A) is currently used to treat numerous medical conditions, including musculoskeletal disorders, dystonia, and pain. The potential use of BoNT/A to treat pain conditions is mainly derived from the observation that patients treated with BoNT/A, e.g. for spasticity or dystonia, often experienced reduction of pain. Although the mechanisms of the analgesic effect of BoNT/A are still not completely elucidated, many preclinical investigations have shown that the analgesic effects may be attributed not only to muscle relaxation but also to the block of transmitters’ release from primary sensory afferent fibers (see [1] for extensive review).

The mode of action of *botulinum* neurotoxins, which are produced in seven serotypes by the bacteria C*lostridium botulinum*, resides on their capacity to cleave synaptic proteins that serve as the basis of neuroexocytosis [2]. Each toxin is composed of a heavy chain (∼100 kDa), which mediates binding to receptors on cell surface, and a light chain (∼50 kDa), which acts as a protease and cleaves the soluble N-ethylmaleimide sensitive factor attachment protein (SNARE) complex that facilitates vesicle fusion and consequent neurotransmitter release [3]–[5]. Among the other *botulinum* neurotoxins, BoNT/A specifically cleaves the 25 kDa synaptosomal-associated protein (SNAP-25), which is anchored to the cell membrane, by removing 9 aminoacid residues at the C-terminus [6]. This cleavage results in a formation of a non-functional SNARE complex thereby blocking the synaptic transmission [7], [8].

The canonical effect of BoNT/A (and of the other serotypes) is mainly exerted as selective inhibitor of evoked acetylcholine (ACh) release from cholinergic nerve endings at the skeletal neuromuscular junction. This effect gave the opportunity to use BoNT/A as therapeutic agent in a variety of neurological disorders due to hyperfunctionality of cholinergic terminals [9]. In the therapy of the hypercholinergic disorders, BoNT/A is locally applied, with little or absent diffusion, and its paralyzing action remains confined to the nerve-muscle junction, close to the injection site. This assumption constituted a “dogma” for many years in the use of these neurotoxins in human therapy. More recently, some experimental evidences from basic scientific research challenge this dogma and raise concern that, while most of the effects are localized to the injection site, BoNT/A can also act distantly from the injection site [10]–[17]. In particular, as far as the effects of BoNT/A on pain are concerned, a number of papers [12]–[15] have demonstrated that the retrograde transport of this toxin has an important role in the analgesic effects in several animal models of pain. However, other authors underline that a number of factors, such as anatomical differences, doses, volume of dilution, etc., have to be taken into account and they suggest a critical attention in the general conclusion shifting from animals to humans [18]–[20].

In previous papers we extensively studied the action of BoNT/A as analgesic in several animal pain models [21]–[24]. In particular, in animal subjected to chronic constriction injury (CCI) of sciatic nerve, a well-established animal model of neuropathic pain, we demonstrated that a single peripheral administration of BoNT/A in the paw of the injured hindlimb was able to counteract pain symptoms and to accelerate functional recovery [23]. Functional recovery was facilitated by an acceleration of regenerative processes associated to the Schwann cells (SC) proliferation. In the present research, we extended our previous result with the aim to prove that retrograde transport of BoNT/A is an important component of the analgesic effects exerted by BoNT/A in neuropathic pain model. We analyzed the effect of intraplantar (i.pl.) injection of BoNT/A (15 pg) on CCI-induced allodynia and on the expression of cleaved-SNAP-25 (cl-SNAP-25) in tissues along nociceptive pathway, from the peripheral nerve endings, to the sciatic nerve, the dorsal root ganglia (DRG) and the spinal cord. The expression of cl-SNAP-25 has been analyzed, both in naïve and neuropathic mice, by immunostaining and confocal microscopy alone or colocalized with glial fibrillary acidic protein (GFAP), a protein marker expressed in sensory nerve endings, in non-myelinating SC [25], and in spinal cord astrocytes [26]. Moreover, we examined colocalization of cl-SNAP-25 with complement receptor 3/cluster of differentiation 11b (CD11b), a protein marker of spinal cord microglia [27], and with neuronal marker (NeuN) in DRG. Finally, in order to better establish possible interaction of BoNT/A with SC, in addition to immunohistochemistry analysis, we performed in vitro experiments to further support the action of BoNT/A and to ascertain if BoNT/A was able to interact with the proliferative state of SC. Some findings of the present research have previously been presented as conference abstract [28].

## Results

### Behavioral Effect of BoNT/A on Neuropathic Pain

The unilateral ligature of the sciatic nerve, as that performed in CCI model of neuropathic pain, induces mechanical allodynia in the hindpaw ipsilateral to the ligature.

**Figure 1:**
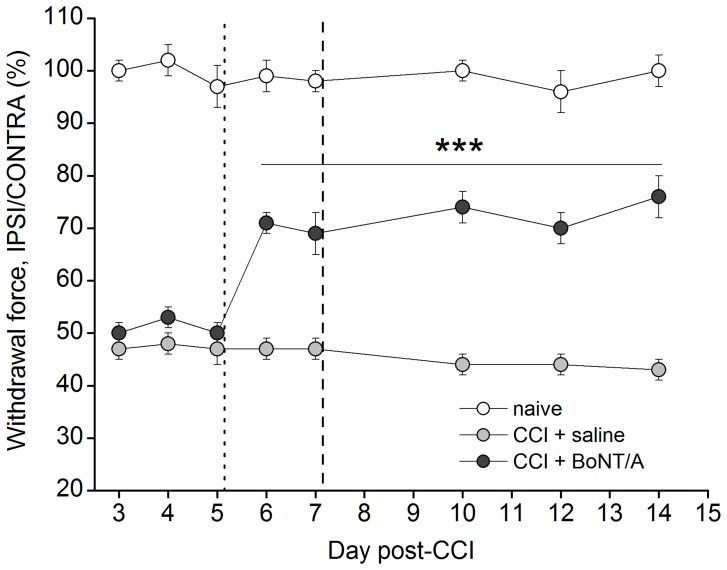
Antagonism by BoNT/A of mechanical allodynia induced by CCI in CD1 mice. Percentage ratio between withdrawal thresholds of hindpaws in naïve (○) mice and between withdrawal thresholds of ipsilateral and contralateral hindpaws in CCI mice intraplantarly injected with saline (grey circle) or BoNT/A (•; 15 pg/paw) into the ipsilateral hindpaw at day 5 from CCI after behavioral measurement (dotted line). Some mice were sacrificed for himmunohystochemistry analysis immediately after behavioral measurement at day 7 from CCI (dashed line). Number of mice were n = 8 for each mice group. Data represent the mean ±SEM. Statistics were done by using Student *t*-test comparison between CCI-BoNT/A and CCI-saline mice (***p<0.001).

The withdrawal thresholds, of both the right and left hindpaws, have been measured and reported in Fig. 1 as the percentage ratio between ipsi- and contralateral withdrawal forces. The withdrawal thresholds in naïve animals, not subjected to CCI, were similar for both hindpaws (12–13 g) and were maintained almost constant for overall the experimental time course. After CCI, the withdrawal threshold decreased of 50% in the ipsilateral compared to contralateral hindpaw. Animals withdrew their ipsilateral paw after very low stimuli (5–6 g), which did not evoke reaction in contralateral paw. For this reason we considered the ipsilateral response as mechanical allodynia. In previous papers [22], [23], under the same experimental condition, we demonstrated that allodynia was maintained for at least 1 month and, even if reduced, it was still present after 3 months. As reported in Fig. 1, a single i.pl. injection of saline, administered 5 days after CCI in the ipsilateral hindpaw, did not alter significantly allodynia. On the other hand, a single i.pl. injection of 15 pg of BoNT/A, administered 5 days after CCI in the ipsilateral hindpaw, markedly antagonized allodynia inducing a clear-cut enhancement of the withdrawal threshold of the ipsilateral hindpaw, with a recovery of 30%. The antiallodynic effect was long-lasting and almost immediate, starting from the day after the injection.

### Immunofluorescence Analysis of cl-SNAP-25 in Paw, Nerve, DRG and Spinal Cord

To establish if the retrograde transport of BoNT/A could explain, at least in part, the observed anti-allodynic effects, we analyzed the expression of cl-SNAP-25 in different tissue, from hindpaw skin to spinal cord. The anti-cleaved SNAP-25 antibody was used to mark the toxin action in distant sites. The cleavage of the BoNT/A substrate SNAP-25 as marker is justified, as already reported [16], by the difficulties to detect the toxin due to very little amount of injected toxin as well as by the fact that a small number of toxin molecules can cleave a massive amount of substrate.

**Figure 2:**
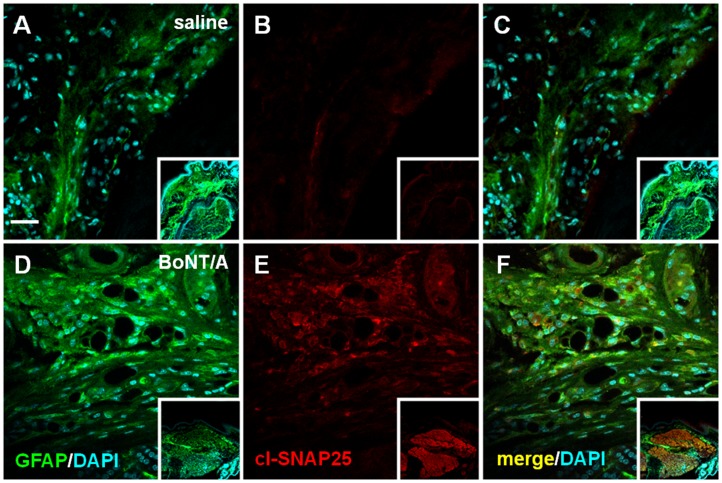
Immunofluorescence detection of cl-SNAP-25 in skin tissue from mice hindpaw. Representative examples of high magnification confocal images taken from section of epidermal hindpaw tissues in naïve mice injected with saline (*panels* **A-C**) or BoNT/A (*panels* **D-F**; 15 pg/paw). In this tissue GFAP (green) is a protein marker expressed in. Insets of each panel represent low magnification confocal images. Scale bar: 20 µM.

Firstly we analyzed the effect of a single i.pl. injection of BoNT/A in control mice not subjected to CCI (naïve mice). Fig. 2 shows representative examples of immunofluorescence (IF) images taken from hindpaw skin sections. In the skin sections of naïve mice i.pl. injected with saline we detected an extensive staining of GFAP (Fig. 2A) accompanied by almost undetectable staining of cl-SNAP-25 (Fig. 2B). Completely different pattern was observed in naïve mice i.pl. injected with BoNT/A. In these mice, the intense GFAP staining in hindpaw nerve endings (Fig. 2D) was accompanied by a diffuse staining of cl-SNAP-25 (Fig. 2E). In particular, high magnification images show a punctuate staining indicating a localization of cl-SNAP-25 in the peripheral nerve terminals.

**Figure 3:**
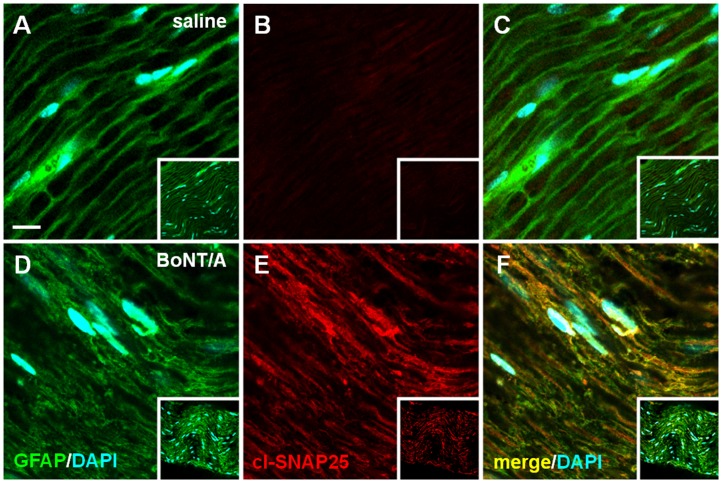
Immunofluorescence detection of cl-SNAP-25 in sciatic nerve. Representative examples of high magnification confocal images taken from section of sciatic nerve in naïve mice injected with saline (*panels* **A-C**) or BoNT/A (*panels* **D-F**; 15 pg/paw). In this tissue GFAP (green) is a protein marker expressed in nonmyelinating SC. Insets of each panel represent low magnification confocal images. Scale bar: 5 µM.

**Figure 4:**
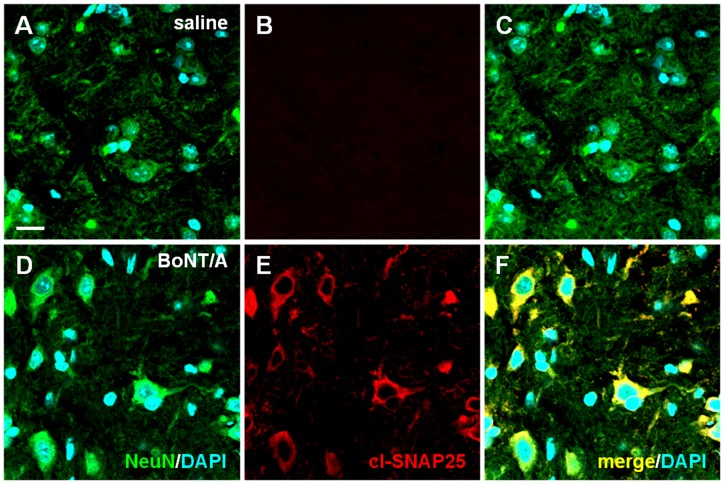
Immunofluorescence detection of cl-SNAP-25 in DRG. Representative examples of high magnification confocal images taken from section of DRG in naïve mice injected with saline (*panels* **A-C**) or BoNT/A (*panels* **D-F**; 15 pg/paw). In this tissue NeuN (green) is a protein marker expressed by neuronal cells. Scale bar: 10 µM.

Fig. 3 shows representative examples of IF images taken from sciatic nerve sections. In sciatic nerve sections of naïve mice i.pl. injected with saline we detected an extensive staining of GFAP (Fig. 3A) while staining of cl-SNAP-25 was almost absent (Fig. 3B). Differently, in nerve sections from naïve mice i.pl. injected with BoNT/A a diffuse staining of cl-SNAP-25 was observed (Fig. 3E), colocalized with staining of GFAP expression (Fig. 3F). In the representative examples of high magnification IF images taken from DRG sections (Fig. 4), as observed in previous figures, the staining of cl-SNAP-25 in naïve mice i.pl. injected with saline was almost undetectable (Fig. 4B), while in naïve mice i.pl. injected with BoNT/A a marked detection of cl-SNAP-25 colocalized with NeuN was observed (Fig. 4E).

**Figure 5:**
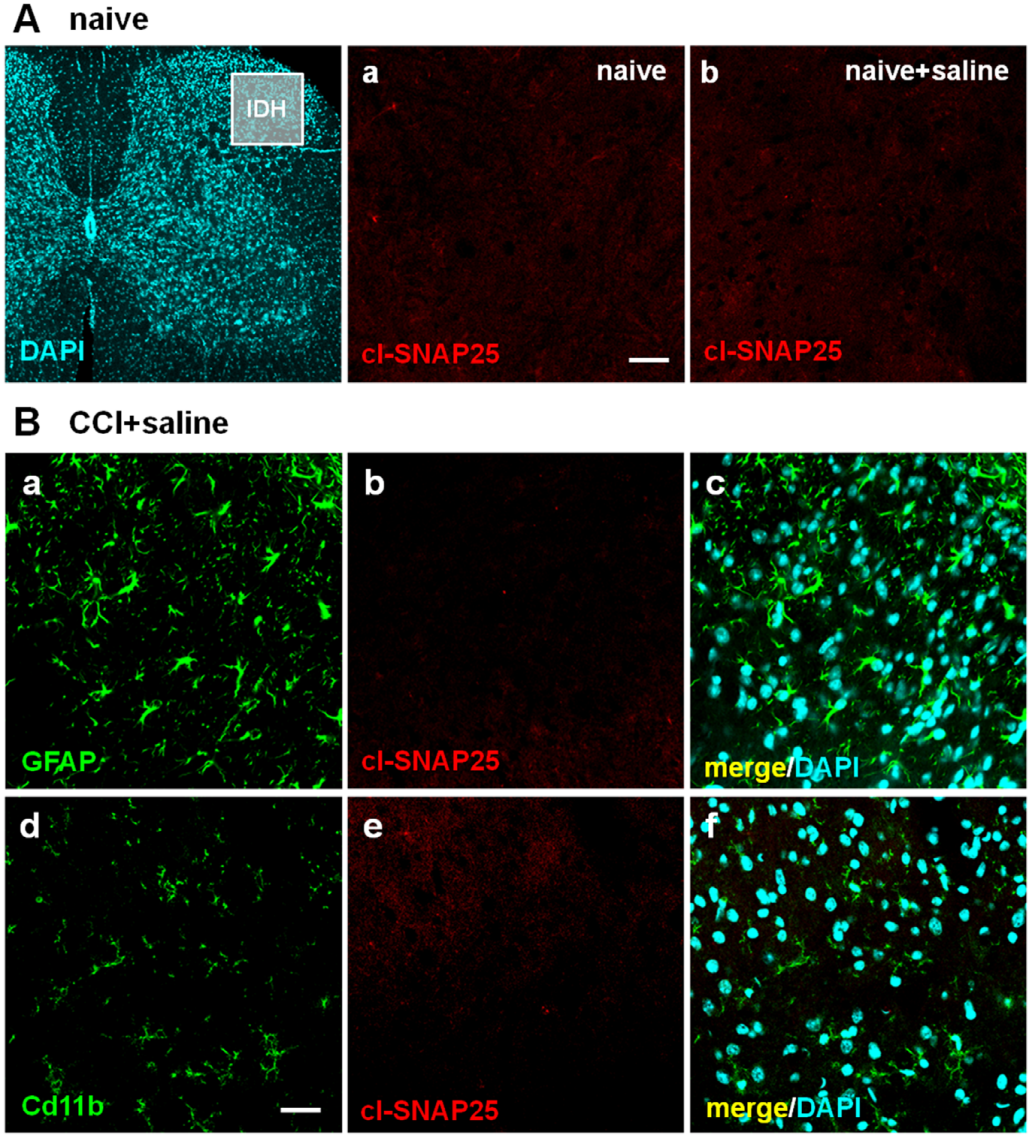
Immunofluorescence detection of cl-SNAP-25 in dorsal horns from mice ipl-injected with saline. A) Representative examples of low and high magnification confocal images taken from lumbar (L4/L5) sections of spinal cord in naïve mice, not subjected to CCI, injected (*panel b*) or not with saline (*panel a*). Dorsal horn region considered for high magnification images is shown by IDH square in the low magnification image. Scale bar: 20 µM. **B)** Representative examples of high magnification images taken form lumbar (L4/L5) sections of spinal cord in CCI mice injected with saline. In these images, GFAP (*panel a*) and CD11b (*panel d*), are protein markers of astrocytes and microglia respectively. *Panels b and e* show immunofluorescence detection of cl-SNAP-25, while *panels c* and *f* show colocalization of cl-SNAP-25 with astrocytes and microglia, and also staining of nuclei with DAPI. Scale bar: 20 µM.

**Figure 6:**
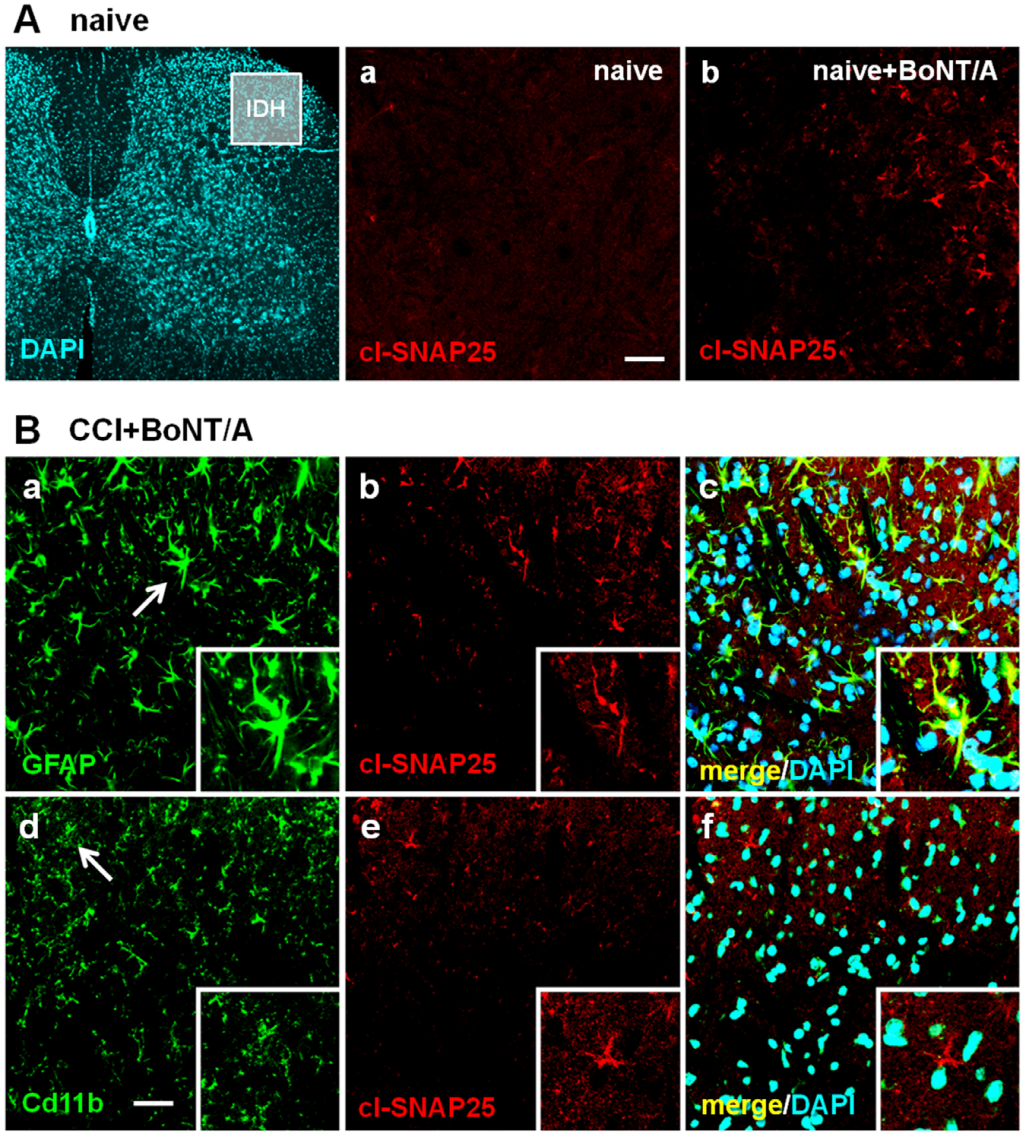
Immunofluorescence detection of cl-SNAP-25 in dorsal horns from mice ipl-injected with BoNT/A. A) Representative examples of low and high magnification confocal images taken from lumbar (L4/L5) sections of spinal cord in naïve mice, not subjected to CCI, injected (*panel b*) or not with BoNT/A (*panel a*; 15 pg/paw). Dorsal horn region considered for high magnification images is shown by IDH square in the low magnification image. Scale bar: 20 µM. **B)** Representative examples of high magnification images taken form lumbar (L4/L5) sections of spinal cord in CCI mice injected with BoNT/A (15 pg/paw). In these images, GFAP (*panel a*) and Cd11b (*panel d*) are protein markers of astrocytes and microglia, respectively. *Panels b* and *e* show immunofluorescence detection of cl-SNAP-25, while *panels c* and *f* show colocalization of cl-SNAP-25 with astrocytes and microglia, and also staining of nuclei with DAPI. Scale bar: 20 µM.

Fig. 5 and 6 show representative examples of IF images taken from ipsilateral L4/L5 spinal cord sections. We analyzed spinal cord section taken both from naïve mice (Fig. 5A and 6A) and from mice subjected to CCI (Fig. 5B and 6B). Fig. 5A shows that the staining of cl-SNAP-25 in spinal cord sections was completely absent in naïve mice, both in the absence (Fig. 5A: *panel a*) and after i.pl. injection of saline (Fig. 5A: *panel b*). In spinal cord sections of mice subjected to CCI, we analyzed the localization of cl-SNAP-25 and its possible colocalization with GFAP in spinal astrocytes and CD11b in microglia. After i.pl. injection of saline in CCI-subjected neuropathic mice (Fig. 5B), we clearly detected staining for astrocytes (Fig. 5B: *panel a*) and microglia (Fig. 5B: *panel d*), but not for cl-SNAP-25 (Fig. 5B: *panels b and e*). A completely different pattern was observed in the presence of BoNT/A. Fig. 6 shows that, after i.pl. injection of BoNT/A in the hindpaw of both naïve (Fig. 6A) and CCI-induced neuropathic mice (Fig. 6B), staining for cl-SNAP-25 in spinal cord sections was clearly detectable (Fig. 6A: *panel b*; Fig. 6B: *panels b and e*). Moreover, after BoNT/A, in CCI-induced neuropathic mice (Fig. 6B), the observed staining for cl-SNAP-25 colocalized with astrocytes (Fig. 6B: *panel c*) but not with microglia (Fig. 6B: *panel f*).

### Effect of BoNT/A on Schwann Cell Proliferation

**Figure 7:**
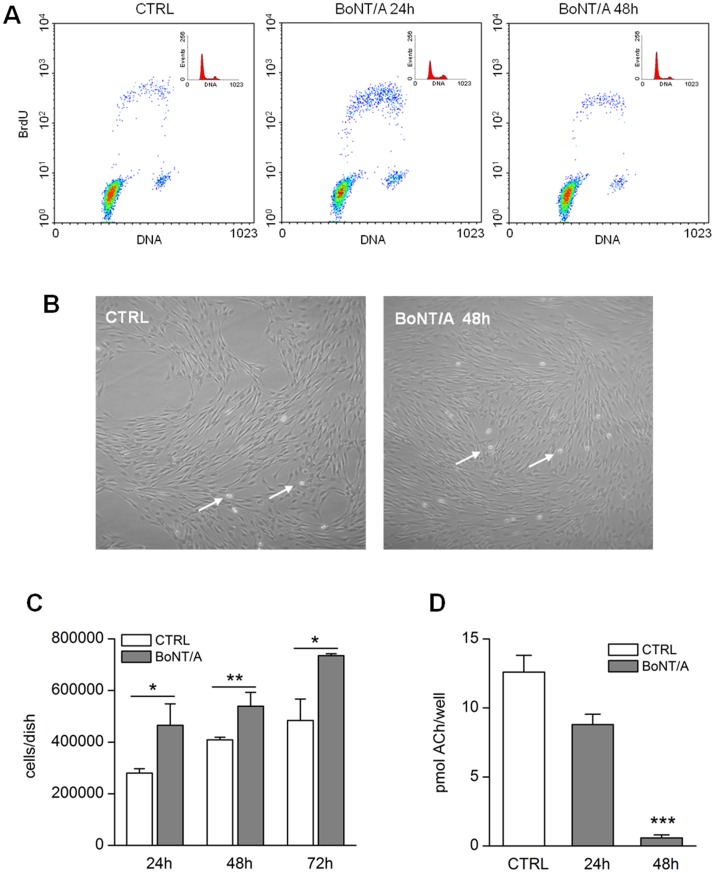
*In vitro* effects of BoNT/A on SC proliferation and ACh release. A) Bivariate analysis of BrdU incorporation and DNA content in SC in absence of BoNT/A treatment (CTRL) and at 24 and 48 h after BoNT/A treatment (10 pM). **B)** Representative images (100X) of cultured SC without (CTRL) or with BoNT/A (10 pM) for 48 h. White arrows indicate proliferating cells. **C)** Counting of the cell number in SC cultures in absence (CTRL) or in presence of BoNT/A for 24, 48, 72 h. Data are the mean ± SEM of three independent experiments performed in duplicate. **D)** Levels of ACh released in culture medium of SC maintained in absence (CTRL) or presence of BoNT/A (10 pM) for 24 and 48 h. Data represent the mean ±SEM of three independent experiments performed in duplicate (***p<0.001).

Marinelli et al. [23] showed that BoNT/A injection in CCI neuropathic mice facilitated functional recovery of injured hindlimb. This probably occurs through an acceleration of regenerative processes since a higher expression of proteins related to structural modifications together with the major number of BrdU-positive proliferative cells in sciatic nerve tissue was observed. To better investigate the BoNT/A action and whether BoNT/A was able to modulate cell proliferation in SC, some in vitro experiments were performed. To establish the effect of BoNT/A on the SC cell cycle, we performed FACS analysis. For this purpose SC were cultured in the presence of 10 pM of BoNT/A for 24 and 48 h. Before harvesting, cell cultures were pulsed with BrdU for 20 min to verify the S phase progression. The bi-parametric analysis of BrdU labeling vs DNA content measured by propidium iodide allowed analysis of cell progression through the G1/S/G2 phases and a more clear identification of cells in S phase. As shown in Fig. 7A, the percentage of SC in S phase significantly increased after 24 h of treatment with BoNT/A compared to untreated cells (CTRL; see also table 1). After 48 h of treatment the percentage of the cells in S phase appeared reduced comparing to 24 h but higher than untreated cells. The mitogenic effect of BoNT/A was also confirmed by the cell counting obtained in cultures in absence or in presence of BoNT/A for 24, 48 and 72h. As reported in Fig. 7C the cell number present in cultures treated with BoNT/A were significantly higher compared to untreated cultures. Some representative images of the cultures in the two different experimental conditions are reported in Fig. 7B. It appears evident that the cultures treated with BoNT/A show a higher cell density/dish.

**Table 1:** Percentage of cells in G1/S/G2 phase obtained from three independent experiments.

Sample	%G1±SEM	%S ±SEM	%G2±SEM
Control	89.26±4.21	6.86±2.94	3.88±1.27
BoNT/A 24 h	73.03±8.30*	18.97±6.95*	7.99±1.44*
BoNT/A 48 h	86.16±1.72	11.89±0.69	4.16±0.37

Statistical significance:

* p<0.05, BoNT/A vs Control.

Considering the ability of the *botulinum* neurotoxins to block the ACh release and since previous data suggested the involvement of ACh in the arrest of SC proliferation [29], we evaluated the ability of SC to release ACh in vitro and the possibility to block this release by BoNT/A. Using fluorimetric assay we have measured the levels of ACh released in the culture medium in presence or absence of BoNT/A. As reported in Fig. 7D, the SC cultured in absence of neurotoxin release in the medium 12.6±1.2 pmol ACh/well. The levels of ACh decreased already after 24 h of BoNT/A treatment (8.8±0.75 pmol ACh/well) and became significantly lower after 48 h (0.58±0.22 pmol ACh/well). These data demonstrate that SC are able to release ACh *in vitro* and that BoNT/A causes a significant decrease of its levels. These results suggest that BoNT/A may enhance SC proliferation counteracting the ACh release.

## Discussion

In the present study we obtained three main findings. First, we demonstrated that peripherally injected BoNT/A may affect neuropathic pain at the central level not only by an indirect but also through a direct action by means of axonal retrograde transport. In details, after i.pl. injection of BoNT/A we observed a significant reduction of mechanical allodynia in CCI-induced neuropathic mice and, associated to these behavioral effects, we detected an extensive staining for cl-SNAP-25 in dorsal horn of spinal cord. As already suggested by other studies [10], [14]–[17], [30], this is a strong indication for the retrograde transport of peripherally injected BoNT/A. Accordingly, cl-SNAP-25 was also found along all the neural nociceptive pathway from the hindpaw skin to the sciatic nerve, the DRG and the spinal cord of naive mice i.pl.-injected with BoNT/A. Second, completely new and surprising result, after peripheral injection of BoNT/A in CCI-neuropathic mice, we detected cl-SNAP-25 in spinal astrocytes. This is a strong indication that BoNT/A may be transcytosed from nociceptive fibers in spinal cord and may enter into glial cells. The third important finding of the present study derives from in vitro experiments where we demonstrated, for the first time, that BoNT/A is able to modulate proliferation of SC by inhibiting their ACh release. Altogether, these results further support the retrograde transport along the nerve and put in evidence completely new biological effects of the toxin. All these findings are particularly important for their intrinsic implications in the knowledge of the action of BoNT/A and for its potential outcome in clinical therapy.

After a pioneering study from Caleo and coworkers, showing that BoNT/A is retrogradely transported by central neurons and transcytosed to afferent synapses [10], an exciting debate on the implications of retrograde transport of BoNT/A in determining its biologic effects developed. Subsequent investigations confirmed that *botulinum* toxins may migrate from the site of injection [11]–[13], [16], [17]. Lackovic and colleagues [14], [30], reporting antinociceptive effects of BoNT/A in formalin-induced facial pain and in infraorbital nerve constriction trigeminal neuropathy, demonstrated that even the analgesic effects of BoNT/A involve axonal retrograde transport of the toxin. In particular, Matak et al. [14] observed a strong detection of cl-SNAP-25 in medullar dorsal horn of trigeminal nucleus caudalis after BoNT/A injection into the whisker pad. The same authors [15] reported immunodetection of cl-SNAP-25 in spinal cord of naïve rats after i.pl. or intramuscular injection of BoNT/A. In the former studies [10], [12], [14]–[17] a passive systemic spread of the toxin was excluded on the basis of evidence that colchicine, the microtubule depolymerizing agent blocking the axonal transport, prevents the effects induced by BoNT/A.

Despite many evidences in favor, the hypothesized retrograde transport of BoNT/A is still a matter of controversy for the scientific community [18]–[20]. Our findings showing that cl-SNAP-25 is detectable into spinal cord astrocytes after i.pl. BoNT/A injection in the hindpaw of CCI-induced neuropathic mice is undoubtedly a further experimental support against a passive diffusion of BoNT/A. Under this point of view, and considering the long distance from peripheral nerve endings to spinal dorsal horns, it can be assumed that BoNT/A is presumably transported by similar axonal trafficking mechanisms as those involved in transport of other pathogens, such as viruses and bacterial toxins [31], [32]. Although the mechanism for axonal transport of BoNT/A remains to be elucidated, it is possible that molecules of BoNT/A, reaching the spinal cord terminal afferents, may undergo transcytosis towards other cells in spinal cord [11].

We have previously proposed [1] that the analgesic effects of BoNT/A may be exerted at different levels, interfering with both peripheral and central sensitization. The present and others studies further support our hypothesis that, even when peripherally injected, a direct central action of BoNT/A may occur. The retrograde transport along axons may be responsible for the displacement of peripheral injected BoNT/A at the level of the spinal dorsal horn where it may exert the inhibition of spinal release of neurotransmitters/neuropeptides, particularly glutamate, from nociceptive neurons in response to nerve injury.

Examining step by step the expression of cl-SNAP-25 after BoNT/A injection in naïve animals, we found a colocalization of the antibody against the cleaved substrate of BoNT/A with GFAP in tissues from hindpaw and sciatic nerve. The colocalization with GFAP, which is expressed in nerve endings of sensory fibers, in unmyelinated fibres of sciatic nerve and in dedifferentiated SC after injury [33], [34], supports the hypothesis that BoNT/A continues its action along the nerve pathway away from the site of injection. It should be reminded that SNAP-25 is not confined only in the axolemma where is involved in synaptic vesicle exocytosis, but it is present along the entire axolemma [35], [36]. Moreover, SNAP-25 labeling was seen in the cytoplasm of the soma and large dendrites, mostly associated with the Golgi complexes. Accordingly, we demonstrated the presence of cl-SNAP-25 in the soma of DRG and, in a previous paper [24], the reduction of SNAP-25 mRNA level in DRG after peripheral administration of BoNT/A. The effects of BoNT/A on the SNARE-dependent release of proalgesic substances from DRG have been extensively studied both *in vitro* and *in vivo*. In cultured embryonic rat DRG, BoNT/A induced a delayed, long-lasting inhibition of SP release following the initial cleavage of SNAP-25 [37]. Also the release of TRPV1 from DRG occurs via SNARE-dependent exocytosis [38]; accordingly, in another study on primary DRG, BoNT/A inhibited the protein kinase C (PKC) mediated SNARE-dependent exocytosis of TRPV1 to the plasma membrane [39]. Kitamura et al., in an *in vivo* study [40], demonstrated that peripheral injection of BoNT/A alleviated the unilateral infraorbital nerve constriction-induced neuropathy behaviors in rats and decreased the exaggerated neurotransmitter release from somata of trigeminal ganglion. More recently, Xiao et al [41] showed that injection of BoNT/A into the plantar surface of the ipsilateral hindpaw significantly inhibited the over-expression of P2X3 receptors in the DRG neurons after selective L5 ventral root transection. Our findings on the presence of cl-SNAP-25 in DRG after BoNT/A injection further support the contribution of BoNT/A on modulation of pain transmission via inhibition of the SNARE-dependent release of proalgesic substances from DRG.

The next step leads to a notably relevant result in our study: when the spinal dorsal horns were examined in neuropathic animals, the colocalization of cl-SNAP-25 with GFAP indicates that the toxin was transcytosed to astrocytes, whose specific marker in spinal cord is GFAP. The role that astrocytes play in mediation of pain, including neuropathic pain, is always growing: these glial cells increase after inflammation and/or nerve injury [42]–[44]. A possible interpretation could be that BoNT/A, transcytosed from neuronal cell to astrocytes, inhibits glutamate release also from astroglial cells and, consequently, contributes to the reduction of pain.

The colocalization of cl-SNAP-25 with GFAP has two considerable new insights that have to be discussed: i) the possibility that BoNT/A is transcytosed to astrocytes and ii) the observation that astrocytes express SNAP-25. Many of neurosecretion-specific proteins were found to be expressed in astrocytes and, even if not by all astrocytes and not in all stages of their life, astrocytes appear to share at least part of the secretion machinery typical of neurons and endocrine cells [45]. In astrocytes cultures, Parpura et al. [46] identified the expression of some of the SNARE protein complex but not of SNAP-25 and Hepp et al. [47] confirmed the absence of SNAP-25, which was replaced by SNAP-23, a homolog present also in microglia and oligodendrocytes. When the effects of *botulinum* neurotoxins were investigated in cultured astrocytes the results resembled those in neurosecretory cells, being glutamate release inhibited although after a long incubation [48]. These authors showed that *botulinum* toxin type B and tetanus toxin cause a decrease in synaptobrevin II immunoreactivity, while they were not able to demonstrate the presence of SNAP-25 (the SNARE protein cleaved by BoNT/A) or syntaxin (the SNARE protein cleaved by BoNT/C) immunoreactivity in cultured astrocytes. On the other hand, pretreatment with BoNT/A and BoNT/C results in a decrease in the baseline release of glutamate and reduces the bradykinin-evoked release of glutamate from cultured astrocytes. Recently, by a comparative analysis of the SNARE proteins in brain tissue from adult mice, Schubert et al. [49] demonstrated that SNAP-25 are absent from astrocytic processes and typically concentrated in terminals, while SNAP-23 have the opposite expression pattern. All these evidences against the expression of SNAP-25 in astrocytes seem to be in contrast with our findings. However, it should be pointed out that our study has not been performed with astrocytes cultures, where in vitro conditions can influence the astroglial properties [50]. Furthermore, it has also been reported that cultured astrocytes express SNAP-25 within few days after plating and show a significant SNAP-25 decline with prolonged culture time [51]. We investigated the expression of SNAP-25 in astrocytes from spinal cord tissues of neuropathic mice, that means animals subjected to a series of complex reactions related to a pathological condition. After lesion of sciatic nerve, a strong proliferation of new astrocytes in spinal cord is observed [43]; this population of proliferating astrocytes may be comparable to the cultured astrocytes within few days after plating. Our finding on the effects of BoNT/A in spinal astrocytes seems to be in contrast also with Matak et al. [15], who were unable to detect cl-SNAP-25 in spinal astrocytes. Again, this apparent discrepancy may be explained considering that authors performed their measurements not in neuropathic but in naïve rats, whose astrocytes population in spinal cord is at adult stage and not in activated state.

Together with the *in vivo* experiments we acquired new important insights with *in vitro* experiments, such as those carried out in SC cultures. Our results show that BoNT/A increased SC proliferation and, for the first time, clearly demonstrate that cultured SC release ACh, as previously suggested by an old study [52]. The inhibition of ACh release from SC by BoNTA is compatible with the possibility that BoNT/A may induce its effects by cleaving SNAP-25 also in SC, opening a new issue in the large variety of biological effects exerted by this versatile molecule. Evidences for the presence of SNAP-25 in SC have been reported by Barden et al. [53] who found that in sympathetic varicosities in mouse vas deferens the protein SNAP-25, together with other SNARE proteins, and N-type calcium channels are clustered with P2X receptors subunits and these receptor clusters are located in SC. On the other hand, the effects of BoNT/A on SC proliferation and ACh release may explain previous observed effects of BoNT/A in the regenerative processes occurring after injury of sciatic nerve [23]. The inhibition of ACh release is a new insight in the functionality of SC, whose proliferation was already associated to ACh by the study of Loreti et al [29], who demonstrate that inhibition of cell proliferation–induced by ACh may depend on negative modulation of the neuregulin 1 (NRG1) levels. In fact, SC proliferation is mainly dependent on levels of NRG1: high levels of NRG1 usually increase SC proliferation and inhibit their differentiation [54]. Although BoNT/A caused a minimal reduction of ACh after 24 h of treatment, this may be sufficient to increase the levels of NRG1 and uncourage Schwann cell proliferation. Thus, our results showing the BoNT/A capacity of facilitating SC proliferation can be explained with the inhibitory action exerted by the neurotoxin on ACh release.

In conclusion, even if further experiments are necessary to fully understand the mechanisms underlying retrograde transport and transcytosis of BoNT/A, our both *ex vivo* and *in vitro* findings, showing that BoNT/A induces analgesic action on neuropathic pain and that exerts its action also through the cleavage of SNAP-25 in astrocytes and on the proliferation of SC after nerve injury, open new exciting view on the large variety of biological effects exerted by this versatile molecules.

## Materials and Methods

### Ethics Statement

This study was carried out in accordance with the recommendations of Italian National law DL116/92 (application of the European Communities Council Directive 86/609/EEC) on care and handling of the animals and with the guidelines of the Committee for Research and Ethical Issues of IASP [55]. The protocol was approved by Italian Ministry of Health (Permit Number: 187/2011-B).

### Materials

CD1 male mice, weighing about 40–45 g, from Charles River Labs (Como, Italy) were used. Mice were housed in standard transparent plastic cages, in groups of 4, lined with sawdust under a standard 12/12-h light/dark cycle (07:00AM/07:00PM), with food and water available ad libitum. BoNT/A (150 KDa purified protein without accessory proteins) was a kind gift from Prof. Cesare Montecucco (Department of Biomedical Sciences, University of Padova, Italy). The toxin was frozen in liquid nitrogen and stored at −80°C in 10 mM NaHepes, 150 mM NaCl, pH 7.2. Stock solutions of BoNT/A were tested for activity in the ex vivo mouse hemidiagphram model and in the *in vitro* cleavage of SNAP-25 [56]. Injectable solutions of BoNT/A were freshly made in saline (0.9% NaCl). Dose of BoNT/A (15 pg/paw) was chosen on the basis of neurotoxicity studies (LD50: 0.5–1.0×10^−6^ mg/kg; [57]) and previous behavioral studies [21]–[23], and it is the maximal effective dose that can be peripherally injected in mice without causing side effects including neuroparalysis.

### Surgery

Following the procedure originally proposed by Bennett and Xie [58], adapted for mice, the Chronic Constriction Injury (CCI) was used as model of neuropathic pain. As previously described [23], CCI was obtained by three unilateral ligatures of sciatic nerve. Anesthesia was performed with 500 mg/Kg (ip) of chloral hydrate (SIGMA-Aldrich, St. Louis, MO). In the paper, injured and uninjured hindlimb will be named as ipsilateral and contralateral, respectively.

### Experimental Groups

Three groups of mice were considered for behavioral analysis: i) naïve mice not subjected to CCI; ii) mice subjected to CCI and injected with saline (0,9% NaCl; 20 µl); iii) mice subjected to CCI and injected with BoNT/A (15 pg/paw; 20 µl). Injection of saline or BoNT/A was subcutaneously administered into the hindpaws’ plantar surface (i.pl.) on day 5 after CCI. All mice were examined for mechanical nociceptive thresholds on day 7 after CCI. The behavioral analysis was performed during the morning (11:00AM/01:00PM) and experimenter was blind as for treatment groups. After behavioral measurements, some mice from each experimental group were sacrificed at day 7 for successive immunohistochemistry analysis. Two additional groups of naïve mice were i.pl. injected with saline or BoNT/A and considered for immunohistochemistry analysis.

### Mechanical Nociceptive Threshold (Dynamic Plantar Aesthesiometer Test)

The onset of neuropathy induced by CCI was assessed by measuring the threshold of both hindpaws to normally non-noxious punctuate mechanical stimuli by using an automatic von Frey apparatus (Dynamic Plantar Aesthesiometer, Ugo Basile, Italy). After 5 min of adaptation of animals to the apparatus, the mechanical stimulus was applied to the midplantar surface of ipsi- and contralateral hindpaw, as previously described [23]. The mechanical threshold is measured as the maximal force (expressed in g) at which mice withdraw their paws. At each testing day, the ipsilateral and contralateral withdrawal thresholds were taken as mean of three consecutive measurements per paw with 10-s interval between each measurement.

### Immunohistochemistry

After sacrifice with a lethal dose of chloral hydrate, the plantar surface of hindpaws, the sciatic nerves and DRG were immediately removed and kept in immersion for 48 h in 4% paraformaldehyde in phosphate buffer saline (PBS, pH 7.4). For immunostaining in spinal cord, mice were anaesthetized with chloral hydrate (500 mg/Kg, ip) and trans-cardiac perfusion was performed with 100 ml saline, followed by 100 ml 4% paraformaldehyde in 0.1 M phosphate-buffered saline (PBS, pH 7.2). After the perfusion, the entire spinal cord of each animal was removed and kept in fixative for 12 h. After cryoprotection with solution of 30% (w/v) sucrose in PBS, all sections were stored at −80°C untill the sectioning. Cryostat microtome sections (20 µm) of hindpaw epidermis, sciatic nerve, and of DRG were taken and mounted directly on glass slides. Transverse sections (40 µm) of L4/L5 spinal cord segment were cut and collected in PBS for free-floating IF procedures. Ipsilateral and contralateral side of spinal cord sections were recognized by marking them with a notch on the contralateral side before mounting spinal cord trunk on the chuck of the cryostat. For double IF staining, sections were first incubated overnight with either anti-GFAP (monoclonal, 1∶100, GenScript Corporation) for immunostaining of nerve endings in hindpaw, nerve fibers in sciatic nerve, and astrocytes in spinal cord, or anti-NeuN (monoclonal, 1∶100, Millipore) as neural marker for DRG, or anti-CD11b (monoclonal, 1∶100, AbD Serotec) for immunostaining of microglia in spinal cord, and anti-cl-SNAP-25 (polyclonal, kind gift of Prof. Ornella Rossetto, University of Padova, Italy) antibodies. After three times washing with PBS, sections were incubated with a mix of goat anti-mouse fluorescein-conjugated (FITC, 1∶100, Jackson ImmunoResearch) and goat anti-rabbit rhodamine-conjugated (TRITC, 1∶100, Jackson ImmunoResearch) secondary antibodies for 2 h at room temperature. Finally, the sections were washed three times in PBS and then incubated with the nuclear marker bisBenzimide, DNA-fluorochrome (Hoechst 33258, 1∶1000, SIGMA-Aldrich) for 5 minutes. Sections were mounted on slides, air dried and cover slipped with glycerol/PBS 3∶1 mounting medium. To exclude non-specific signals of secondary antibodies and to ensure optimal results, control sections have been stained with secondary antibody alone, which did not show appreciable staining (data not shown).

All the immunofluorescence images of paw, sciatic nerve, DRG, and spinal cord were obtained by laser scanning confocal microscopy using a TCS SP5 microscope (Leica Microsystem). All analyses were performed in sequential scanning mode to rule out cross-bleeding between channels. High magnification (63×) images of different sections were acquired at different zoom depending on different tissue analyzed and operated by I.A.S. software (Delta Systems, Italy). Figures were assembled by using Adobe Photoshop CS3 and Adobe Illustrator 10.

### Cell Cultures

Schwann cells were obtained from sciatic nerves according to the method described by Brockes [59] and modified by Davis and Stroobant [60] (see also [29]). The nerves were digested for 15 min at 37°C in 1% collagenase (Type I, SIGMA-Aldrich) and 2.5% trypsin (Gibco). At the end of the proteolytic treatment, the suspension was diluted with fresh DMEM containing 25 mM Hepes and 10% fetal calf serum (FCS, SIGMA-Aldrich) and the nerves dissociated. The resulting cell suspension was filtered through a 63-mm pore nylon mesh and centrifuged at 1000 rpm for 5 min. Cells were then cultured in flasks with DMEM plus 10% FCS, at 37°C in a humidified atmosphere of 10% CO2/90% O2. After 24 hours, the medium was replaced with fresh medium containing the antimitotic agent cytosine arabinoside (AraC, final concentration 1 mM) to inhibit fibroblast proliferation. Two days later, AraC was removed and the cells washed with fresh medium and DMEM containing 10% FCS, 5 mM forskolin (Fsk, SIGMA-Aldrich) and bovine pituitary extract (SIGMA-Aldrich, final dilution 1∶150) was added. Once confluent, cells were removed from the flask with 0.05% trypsin in phosphate-buffered saline (PBS) containing 0.02% EDTA, washed, and treated with anti-Thy-1.1 (1∶1000, AbD Serotec) and rabbit complement (1∶2 v/v) (Cederlane) to remove residual fibroblasts. Schwann cells were amplified in DMEM, 10% FCS, 5 µM Fsk and bovine pituitary extract (1∶150) and maintained in DMEM, 10% FCS, 2 µM Fsk during experiments.

### Analysis of Cell Number

Schwann cells were plated onto dishes (35-mm diameter) at density 5×10^4^ cells/dish and maintained for 24, 48 and 72 h in DMEM plus 10% FCS, 2 µM Fsk either in the presence or absence of BoNT/A (10 pM). Cells were then collected by trypsinization and counted using the Bürcker chamber.

### Flow Cytometry Analysis

The cells were plated onto 90-mm diameter dishes at a density of 1×10^6^ cells/dish. The day after plating the cells, excluded control samples were treated with BoNT/A (10 pM) for 24, 48 h. At the end of the treatments, cells were incubated for 20 min with 45 µM bromodeoxyuridine (final concentration) (BrdU, SIGMA-Aldrich), collected by trypsinization, centrifuged for 10 min at 1000 rpm and then fixed in methanol/PBS 1∶1 (v/v). In order to identify cells in the S phase, DNA content and BrdU incorporation were determined in simultaneous analysis by staining with propidium iodide (PI) and fluorescein isothiocyanate (FITC)-conjugated anti-BrdU antibody (Vector Laboratories) respectively. Partial DNA denaturation was performed incubating the cells in 3N HCl for 45 min, followed by neutralization with 0.1 M sodium tetraborate. Samples were washed with 0.5% Tween-20 in PBS then incubated with a mouse monoclonal anti-BrdU antibody (1∶50, Dako) for 1 hour at room temperature, washed twice with 0.5% Tween-20 in PBS and incubated for 45 min with Alexa Fluor 488-conjugated anti mouse antibody (Molecular Probes, Eugene, OR). Samples were washed twice with PBS and finally stained with 10 µg/ml PI for 15 min at room temperature. Flow cytometry analysis was performed with a flow cytometer Coulter Epics XL with 488 nm wavelength excitation and 10^4^ events were collected for each sample. Monoparametric (DNA histograms) and biparametric (DNA content vs BrdU content) analysis were performed using WinMDI 2.7 software.

### Acetylcholine Assay

ACh was measured by commercial colorimetric/fluorimetric kit (Abcam, Cambridge, UK) (see [61]). Fifty µl of the sample were mixed with 50 µl of reaction solution including choline assay buffer, choline probe, enzyme mix and AChE according to kit’s instructions. The level of Ch/ACh (pmol/well) was calculated plotting the fluorescence of each sample in relation to choline standard curve. The standard curve has been obtained diluting the choline standard according to fluorimetric procedure as indicated by manufacturer’s instructions. The concentrations of the standard choline that have been plotted were: 10–20–30–40–50 pmol. The measurement of the fluorescence was obtained by Glomax Multi Detection System (Promega) at λ Ex/Em 535/587 nm.

### Statistical Analysis

Statistical significance were evaluated by Student’s *t*-test. Data were considered statistically significant at p<0.05 (*), p<0.01 (**) and p<0.001 (***).
